# Systematic review of the molecular basis of hereditary breast and ovarian cancer syndrome in Brazil: the current scenario

**DOI:** 10.1186/s40001-024-01767-x

**Published:** 2024-03-20

**Authors:** Andreza Amália de Freitas Ribeiro, Nilson Moreira Cipriano Junior, Luciana Lara dos Santos

**Affiliations:** Universidade Federal de São João del Rei (UFSJ), 400 Sebastião Gonçalves Coelho St, Divinópolis, MG 35501-296 Brazil

**Keywords:** HBOC in Brazil, Hereditary breast and ovarian cancer, *BRCA* mutations, Systematic review

## Abstract

**Background:**

A detailed understanding of the genetic basis of cancer is of great interest to public health monitoring programs. Although many studies have been conducted in Brazil, a global view on the molecular profile related to hereditary breast and ovarian cancer (HBOC) in this large and heterogeneous population is lacking.

**Methods:**

A systematic review following the PRISMA guidelines was conducted in three electronic databases (PubMed, BIREME and SciELO). Brazilian studies covering molecular analysis of genes related to HBOC, published until December 2023, were considered.

**Results:**

We identified 35 original studies that met all the inclusion criteria. A total of 137 distinct mutations were found in the *BRCA1* gene, but four of them corresponded to 44.5% of all mutations found in this gene. The c.5266dupC *BRCA1* mutation was responsible for 26.8% of all pathogenic mutations found in the *BRCA1* gene in patients with clinical criteria for HBOC from the Brazilian population. Considering all studies that track this mutation in the *BRCA1* gene, we found a frequency of 2% (120/6008) for this mutation in Brazilian patients. In the *BRCA2* gene, the four most frequent mutations corresponded to 29.2% of pathogenic mutations. Even though it was tracked by few studies, the c.156_157insAlu mutation was responsible for 9.6% of all pathogenic mutations reported in the *BRCA2* gene. Seventeen studies found pathogenic mutations in other non-*BRCA* genes, the c.1010G > A mutation in the *TP53* gene being the most frequent one. Considering all studies that screened for this specific mutation in patients with the clinical criteria for HBOC, the frequency of c.1010G > A was estimated at 1.83% (61/3336).

**Conclusions:**

Despite significant molecular heterogeneity among mutations in HBOC patients from Brazil, three mutations deserve to be highlighted, c.5266dupC, c.156_157insAlu and c.1010G > A in the *BRCA1*, *BRCA2* and *TP53* genes, respectively. With more than 200 records, these three mutations play a vital role in the pathology of breast and ovarian cancer in Brazil. The data collected shed light on the subject, but there is still not enough data from certain subpopulations.

**Supplementary Information:**

The online version contains supplementary material available at 10.1186/s40001-024-01767-x.

## Introduction

Hereditary breast and ovarian cancer (HBOC) accounts for 5–10% of all breast cancer (BC) cases and is inherited in an autosomal dominant manner. The large number of cases of HBOC syndrome are caused by the presence of germline mutations in either the *BRCA1* or the *BRCA2* gene [1]. Women with pathogenic *BRCA1/2* variants have up to an 87% risk of developing an associated cancer, while men have up to a 20% risk [2]. However, not all cases of HBOC can be assigned to *BRCA1* and *BRCA2*, as more than 20 other genes have been associated with an increased risk of familial breast and/or ovarian cancer [3].

In Brazil, the estimated incidence of breast cancer was 73.610 new cases for 2023. Considering cases by region, the estimate for breast cancer in women was higher in the Southeast (84.46/100 thousand inhabitants), followed by the South (71.44/100 thousand inhabitants), Midwest (57.28/100 thousand inhabitants), Northeast (52.20/100 thousand inhabitants) and North (24.99/100 thousand inhabitants). Ovarian cancer is less frequent than breast cancer, with 7.310 new cases estimated for 2023. However, it is one of the most common cancers in Brazil. The regions of Brazil with the highest gross incidence rates per 100,000 inhabitants are the South, followed by the Southeast and Northeast, with lower rates in the North and Midwest regions [4].

The risk of developing breast and ovarian cancer may differ according to the mutations present in the individual’s genes, as well as their nationality and family history. Even though *BRCA* mutations occur in all ethnic groups, their prevalence varies among different countries and diverse groups within the same country [5]. Recognition of a genetic predisposition to cancer, the preventive measures available to healthy women with a *BRCA* mutation and the personalized cancer therapies available for *BRCA*-positive patients have reinforced the indication for *BRCA* testing worldwide. Currently, clinical and genetic analyses can guide the choice of treatment and the selection of the combination of antineoplastic agents to be used in a specific case [6].

Genetic testing remains expensive and inaccessible for most women in developing countries. A limited number of studies in the Brazilian population have focused on *BRCA* sequencing or screening for specific mutations in some HBOC-related genes. With the advent of next-generation sequencing (NGS) and the possibility of evaluating a panel of genes related to cancer simultaneously, the number of studies with this aim has increased in the Brazilian population [7]. However, there are still few studies, and most of them are restricted to a specific region of the country [6, 8]. Despite all this diversity, the only study reporting a more representative profile of HBOC mutations in Brazil was performed by laboratory reports showing pathogenic or likely pathogenic germline mutations from tests performed for *BRCA1* and *BRCA2* from several public and private health located in different Brazilian states [9].

The Brazilian population is one of the most heterogeneous in the world and originated from three major ethnic groups: Portuguese, Africans and Amerindians. Other minor groups also contributed to the genetic plurality of the country [10]. As the pattern of ethnic diversity in Brazil is complex, the objective of this review is to provide an update on the molecular basis of the HBOC syndrome in a very diverse population that differed in the occupation process during the time of colonization and to demonstrate the geographic variability of the genetic mutations described within the country.

## Methods

This systematic review was reported according to the Preferred Reporting Items for Systematic reviews and Meta‐analysis (PRISMA) guidelines [11]. Study selection was conducted in three phases: identification, screening and eligibility. Two independent researchers performed the initial study identification by searching three databases, PubMed, SciELO and BIREME. The bibliographic search included all studies published until december 2023. The descriptors HBOC AND Brazil, Hereditary Breast and Ovarian Cancer AND Brazil and *BRCA* mutations AND Brazil were cross-checked with each database. The inclusion criteria were primary articles covering molecular analysis of genes related to HBOC in Brazil in unrelated patients with breast and/or ovarian cancer or HBOC patients. The exclusion criteria were primary articles not covering molecular analysis related to HBOC genes in Brazil, review articles, studies that did not report the variants found, those which used non-Brazilian populations, and those articles that studied only somatic mutations. A systematic review flowchart was developed following the PRISMA specifications.

In the screening phase, all duplicate articles and those in which the title or subject of the abstract was outside the study objective, were excluded. In the eligibility phase, articles where all the full content was outside the main objective were excluded. To reduce bias in the data analysis, an email was sent to authors with similar works. The articles were analysed by two different evaluators, and those that met the inclusion criteria were selected for this review.

The Human Genome Variation Society (HGVS) nomenclature was followed despite some articles describing the mutations with different nomenclatures and the reference transcripts for each mutation can be accessed in ClinVar database.

## Results

**Fig. 1:**
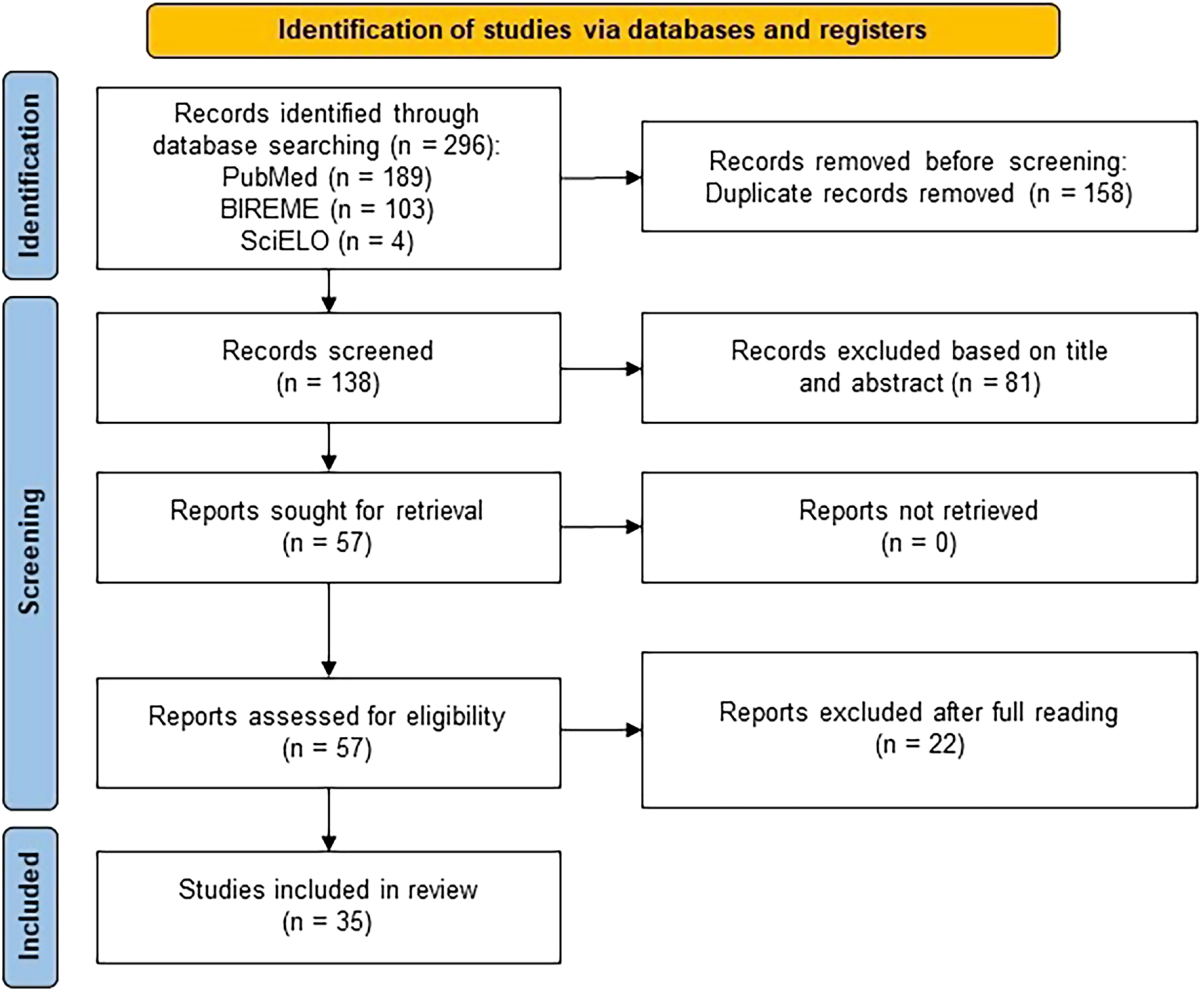
Flowchart representing the process of screening and selection of eligible studies, based on Preferred Reporting Items for Systematic Reviews (PRISMA) guidelines [11]

The database search generated a total of 296 records (Fig. 1). Of these, 158 were excluded after checking for duplication between databases. Our literature search returned 136 records from the three databases, and two articles were added from other sources (e.g., cross-references), resulting in 138 records. Eighty-one articles were excluded on the basis of their titles and abstracts. We evaluated the full text of 57 studies considered potentially eligible by the two authors and, after analysis, 22 manuscripts were removed because they did not meet the inclusion criteria. In the end, 35 studies were eligible for qualitative synthesis.

**Fig. 2:**
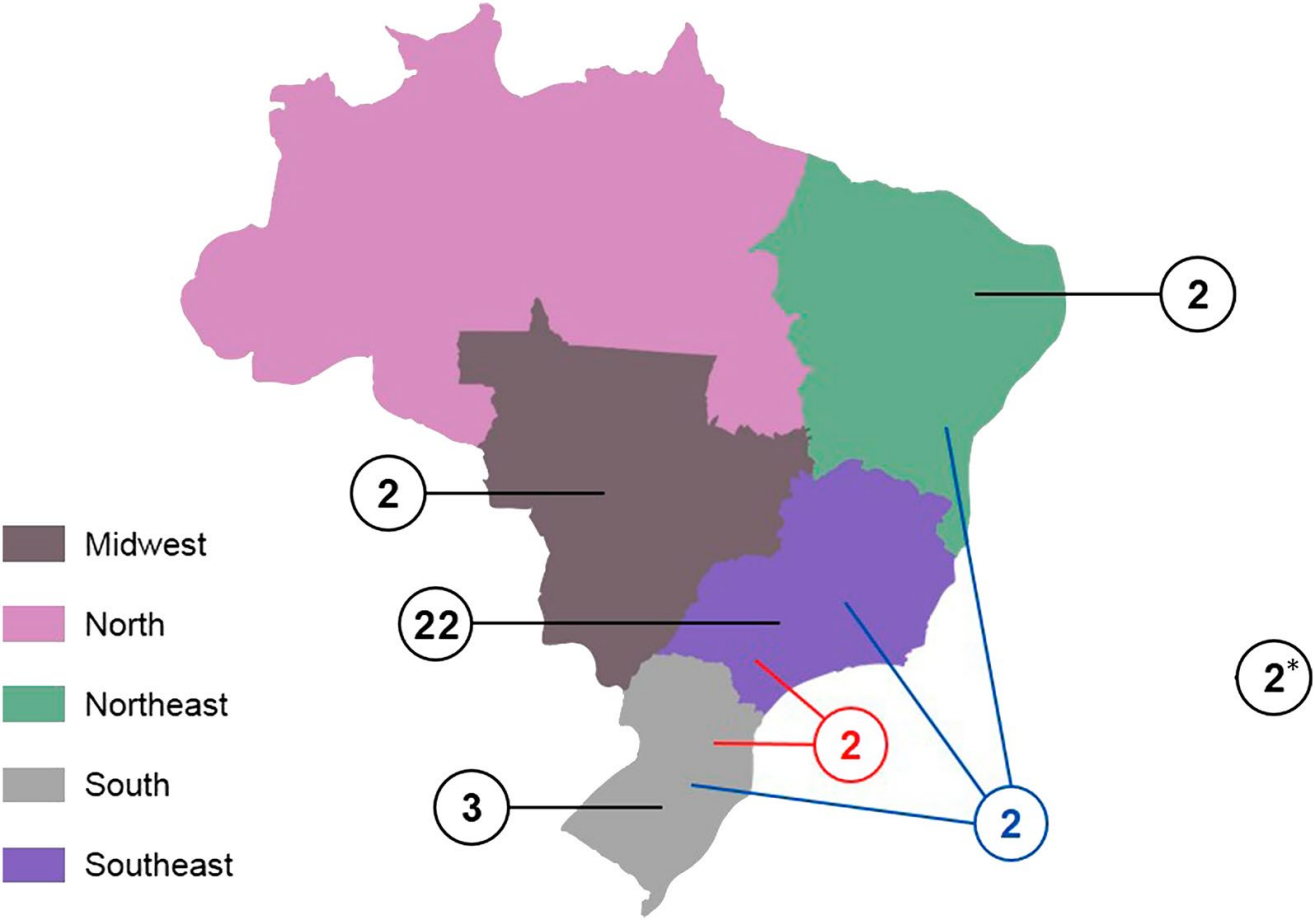
Geographical distribution of the studies included in this review according to the regions of Brazil. Each black line indicates the number of studies with samples from the respective region of the country (they are not distributed on the map according to the state or municipality localization). Red lines indicate the number of studies with samples from South and Southeast regions. The blue lines indicate studies that used populations from more than two regions (South, Southeast and Northeast). *Only two study included samples from all regions of the country

According to the Brazilian regions studied, most of the articles described HBOC populations in the Southeast and South regions of the country, with few studies in the Northeast and Midwest (Fig. 2). Most of the patients came from the states of São Paulo, Rio de Janeiro and Rio Grande do Sul. However, it is not possible to obtain information about the place of birth for most of the patients included in the studies. Therefore, data on geographic location should be interpreted with caution.

**Fig. 3:**
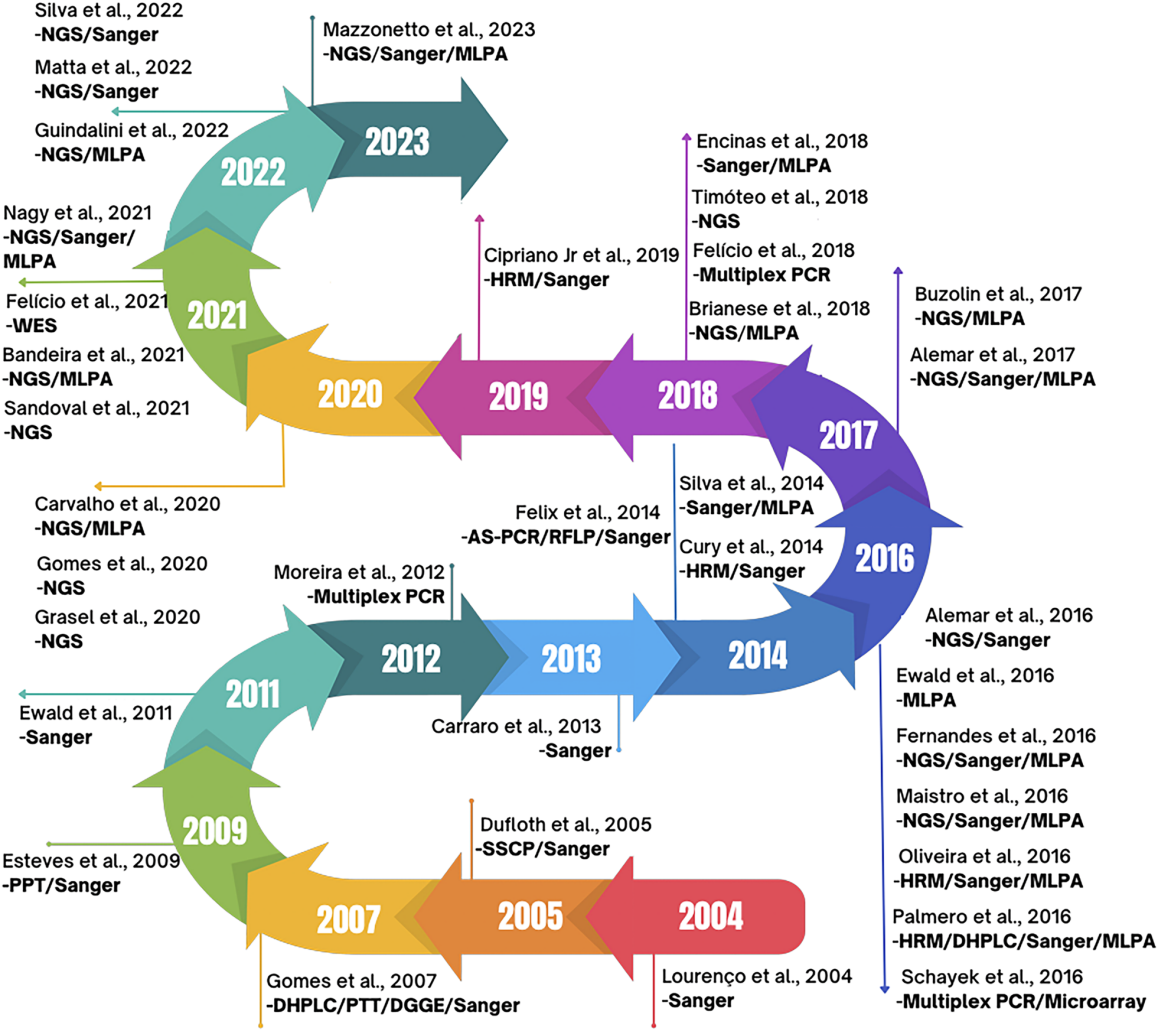
Timeline showing the distribution of Brazilian studies and all methods used to track mutations in HBOC populations

When analyzing the distribution of articles per year, we found that the first Brazilian molecular evaluation study for HBOC was conducted in 2004. Ten articles were published in the next decade and Sanger sequencing was the most used technology. Several other techniques were employed, alone or in association with others, such as: Single-Stranded Conformation Polymorphism (SSCP), Denaturing Gradient Gel Electrophoresis (DGGE), Protein Truncation Test (PPT), Restriction Fragment Length Polymorphism (RFLP), Denaturing High Performance Liquid Chromatography (DHPLC), Allele-specific PCR (AS-PCR), multiplex PCR, High Resolution Melting (HRM), Real-time PCR (qPCR), Microarray and Multiplex Ligation-dependent Probe Amplification (MLPA). The first articles using NGS were from 2016 and in 2018 the first multigene analysis for non-BRCA genes was performed. From this date to now the NGS methodology has stood out as the method of choice. A timeline with all the articles identified, as well as the methods used, can be seen in (Fig. 3).

Among the 35 papers reviewed, 18 screened the complete *BRCA* genes, four using NGS technology only for *BRCA* genes, four using Sanger sequencing, four using both methodologies and the last six studies using NGS panels including *BRCA* genes. Only two studies performed NGS panel in which *BRCA* genes were not included and one was performed using next generation sequencing just for *TP53* gene. There were three studies that tracked only the complete *BRCA1* gene with or without analysis of point mutations in other genes, and nine studies used various techniques for tracking specific mutations or analyzing isolated exons. Only one study performed exome analysis and another one, only the MLPA technique. It is important to emphasize that among all the studies mentioned above, 15 of them also analyzed rearrangements in *BRCA* genes by MLPA.

Twenty-nine studies which examined *BRCA* mutations were verified, 22 of which reported finding mutations in both the *BRCA1* and *BRCA2* genes. Among 18 studies where whole *BRCA* genes were sequenced, the frequency of mutations in the *BRCA1* gene was considerably higher than that in *BRCA2* in 16 of them. Seventeen articles describing mutations in genes other than *BRCA* were found, and the majority of mutations were in the *TP53*, *MUTYH*, *ATM*, *PALB2* and *CHEK2* genes.

The description of the pathogenic mutation frequency found in each gene distributed by region of the country is shown in Table 1. Variants of unknown significance (VUS) were not included in this review.

**Table 1:** Prevalence of pathogenic mutations in HBOC susceptibility genes in Brazilian studies

Region of Brazil	State	Method	Population based on clinical criterias	n	VUS	% pathogenic mutations	% *BRCA1 *mutations	% *BRCA2* mutations	% other genes mutations	Other genes mutated	References
Northeast	RN	NGS panel including *BRCA* genes	HBOC	157	4	23/157 (14.6%)	11/157 (7.0%)	5/157 (3.1%)	7/157 (4.4%)	*ATM*/*ATR*/*CDH1*/*MLH1*	Timóteo et al. [12]
Northeast	BA	AS-PCR/RFLP/Sanger sequencing for complete *BRCA1*/point mutations in *BRCA2*, *TP53* and *CHEK2*	HBOC	106		10/106 (9.4%)	9/106 (8.5%)	0/106 (0%)	1/106 (0.9%)	*TP53*	Felix et al. [13]
South	RS, SC and PR	Sanger sequencing and NGS for complete *BRCA* genes/ MLPA	HBOC	418	24	83/418 (19.8%)	51/418 (12.2%)	32/418 (7.6%)			Alemar et al. [14]
South	RS	Sanger sequencing and NGS for complete *BRCA* genes	HBOC	193		44/193 (22.8%)	26/193 (13.4%)	18/193 (9.3%)			Alemar et al. [15]
		*BRCA* mutations Mass ARRAY MALDI-TOF panel	HBOC	232		8/232 (3.5%)	6/232 (2.6%)	2/232 (0.8%)			
South	RS	DHPLC/HRM and sanger sequencing for *BRCA* genes/ MLPA	HBOC	18	5			1/18 (5.5%)			Palmero et al. [16]
		HRM/Sanger sequencing for 2 *TP53* exons	LFL	40	2						
		Sanger sequencing for *CHEK2* 1100delC mutation	HBCC	7					1/7 (14.2%)	*CHEK2*	
Southeast	SP	NGS for complete *BRCA* genes/ MLPA	HBOC	94	10	10/94 (10.7%)	5/94 (5.3%)	5/94 (5.3%)			Buzolin et al. [17]
Southeast	SP	Sanger sequencing for complete *BRCA* genes/point mutation in *TP53* and *CHEK2* genes/MLPA and array-CGH for other genes	HBOC	120	19	31/120 (26%)	20/120 (16.7%)	7/120 (5.8%)	4/120 (3.3%)	*TP53* / *CHEK2*	Silva et al. [18]
Southeast	SP	Sanger sequencing and NGS for complete *BRCA* genes/*TP53* for 274 patients/MLPA	HBOC	349	36	75/349 (21.5%)	49/349 (14.0%)	26/349 (7.4%)	9/274 (3.3%)	*TP53*	Fernandes et al. [19]
Southeast	SP	NGS for complete *BRCA* genes/MLPA	Triple negative Breast Cancer	131	7	18/131 (13.7%)	16/131 (12.2%)	2/131 (1.5%)			Brianese et al. [20]
Southeast	SP	HRM/Sanger sequencing for *TP53* p. R337H mutation	HBOC	28		2/28 (7.1%)			2/28 (7.1%)	*TP53*	Cury et al. [21]
Southeast	SP	WES for non *BRCA *mutation carriers	HBOC	52	92	12/52 (23%)			12/52 (23%)	*CHEK2*/*RAD51C*/*PMS2*/*DROSHA*/*HERPUD1*/*PLK2*/*RIPK1*/*CTNNA1* /*LCP1 PTCH1*/*CTNNA2*	Felício et al. [22]
Southeast	SP	NGS panel including *BRCA* genes/ MLPA	HBOC	105	15	17/105 (16.2%)	10/105 (9.5%)	3/105 (2.8%)	4/105 (3.8%)	*ATM*/*TP53*/*RAD51*/*BIRP1*	Bandeira et al. [23]
Southeast	SP	Non *BRCA* NGS panel	HBOC	52	11				2/52 (3.8%)	*MUTYH*/*MRE11A*	Grasel et al. [24]
Southeast	SP	Sanger sequencing or NGS for complete *BRCA* genes and MLPA	Ovarian cancer	100	19	19/100 (19%)	17/100 (17.0%)	2/100 (2.1%)			Maistro et al. [25]
Southeast	SP	SSCP and sanger sequencing for four *BRCA1* exons and three *BRCA2* exons	HBOC	31	1	3/31 (9,67%)	1/31 (3.2%)	2/31 (6.4%)			Dufloth et al. [26]
Southeast	SP	Sanger sequencing for complete *BRCA* genes/*TP53* and *CHEK2* c.1100delC	HBOC	54	12	12/54 (22.2%)	7/54 (12.9%)	4/54 (7.4%)	1/54 (1.8%)	*TP53*	Carraro et al. [27]
Southeast	SP	NGS panel/ MLPA	HBOC	95	72	22/95 (23.4%)	13/95 (13.6%)	4/95 (4.2%)	5/95 (5.2%)	*TP53*	Carvalho et al. [7]
Southeast	SP	Sanger sequencing or NGS for complete *BRCA* genes and MLPA	HBOC	49	8	5/49 (10.2%)	3/49 (6.1%)	2/49 (4.1%)			Nagy et al. [28]
Southeast	SP	Sanger sequencing for complete *BRCA* genes/MLPA	HBOC	79	29	13/79 (16.5%)	4/79 (5.1%)	9/79 (11.4%)			Encinas et al. [29]
Southeast	MG	Microarray for pontual *BRCA* mutations, qPCR for *TP53* p. R337H/Multiplex PCR for c.156_157insAlu *BRCA2*	Ovarian cancer	103		0/103 (0%)					Schayek et al. [30]
Southeast	MG	HRM and Sanger Sequencing for *BRCA* genes/Sanger Sequencing for *TP53* p. R337 and *CHEK2* 1100delC mutations	HBOC	44	1	13/44 (29.5%)	5/44 (11.3%)	7/44 (15.9%)	1/44 (2.2%)	*TP53*	Cipriano et al. [31]
Southeast	MG	HRM and Sanger Sequencing for *BRCA1*/MLPA	HBOC	18		2/18 (11.1%)	2/18 (11%)				Oliveira et al. [32]
Southeast	RJ	Multiplex PCR for c.156_157insAlu *BRCA2*	HBOC	168		3/168 (1.8%)		3/168 (1.8%)			Moreira et al. [33]
Southeast	RJ	Sanger sequencing for complete *BRCA1* gene	HBOC	47		7/47 (15%)	7/47 (15.0%)				Lourenço et al. [34]
Southeast	RJ	DHPLC/PTT/DGGE/Sanger sequencing for pontual mutations in *BRCA* genes	Breast cancer	402		9/402 (2.3%)	6/402 (1.5%)	3/402 (0.7%)			Gomes et al. [35]
Southeast	RJ	Non *BRCA* NGS panel	HBOC	126	8	3/126 (2.4%)			3/126 (2.4%)	*ATM* / *PALB2*/ *TP53*	Gomes et al. [36]
Southeast	RJ	NGS panel/Sanger sequencing	HBOC	257	27	39/257 (15.2%)	18/257 (7%)	15/257 (5.8%)	6/257 (2.3%)	*TP53*/ *ATM*/* BARD1*	Matta et al. [37]
Midwest	DF	NGS panel/*BRCA1/2* sequencing	HBOC (85.3%)	224	140	39/224 (17.5%)	12/224 (5.4%)	6/224 (2.7%)	21/224 (9.4%)	*ATM*/ *BARD1*/ *MUTYH*/ *MSH6*/ *PALB2*/ *RAD51C*/ *RAD51D*/ *RECQL4*/* TP53*	Sandoval et al. [38]
Midwest	GO	NGS for *TP53*	HBOC	83		4/83 (4.8%)			4/83 (4.8%)	*TP53*	Silva et al., [39]
Southeast, South and Northeast	RS, RJ and BA	MLPA for *BRCA*	HBOC	145		5/145 (3.4%)	2/145 (1.4%)	3/145 (2.0%)			Ewald et al. [40]
Southeast and South	RS and RJ	Sanger sequencing for 3 *BRCA1* exons	HBOC	137		7/137 (5.1%)	7/137 (5.1%)				Ewald et al. [41]
Southeast and South	SP, RS and RJ	Multiplex PCR for c.156_157insAlu *BRCA2*	HBOC	1380		9/1380 (0.65%)		9/1380 (0.65%)			Felício et al. [42]
Southeast, South and Northeast	SP, PI, RJ and RS	Sanger sequencing/PPT for *BRCA1* exon 11 and *BRCA2 *exons 10 and 11	HBOC	612		21/612 (3.4%)	18/612 (2.9%)	3/612 (0.5%)			Esteves et al. [43]
All regions	-	NGS panel/ MLPA	HBOC	1663	775	278/1663 (16.7%)	92/1663 (5.5%)	64/1663 (3.8%)	122/1663 (7.3%)	*ATM*/ *BARD1*/ *BLM*/ *BRIP1*/ *CHEK2*/ *FANCC*/ *MLH1*/ *MSH2*/ *MUTYH*/ *NBN*/ *PALB2*/ *RAD51C*/ *RAD51D*/* TP53*	Guindalini et al. [44]
All regions	-	Sanger sequencing or NGS for complete *BRCA* genes/MLPA	HBOC	1267	31	156/1267 (12,3)	93/1267 (7,3%)	63/1267 (5%)			Mazzonetto et al. [45]

*BA* Bahia, *DF* Distrito Federal, *GO* Goiás, *HBCC* Hereditary breast and colorectal cancer, *HBOC* Hereditary breast and ovarian cancer, *LFL* Li-Fraumeni Syndrome, *MG* Minas Gerais, *PI* Piauí, *PR* Paraná, *RJ* Rio de Janeiro, *RN* Rio Grande do Norte, *RS* Rio Grande do Sul, *SC* Santa Catarina, *SP* São Paulo, *VUS* variants of unknown significance

A database was created with all pathogenic mutations identified in the reviewed articles (Additional file 1). Studies that reported the use of the same sample in two different studies were excluded. Considering all the works included in this review, a total of 137 distinct mutations were found in the *BRCA1* gene (Additional file 1), but four of them (Table 2) correspond to 44.5% of all pathogenic mutations in this gene in the Brazilian population. In the *BRCA2* gene, 127 different mutations were identified (Additional file 1) and the four most frequent mutations corresponded to 29.2% of pathogenic mutations in this gene (Table 2). The most common mutations in *BRCA* genes found in the Brazilian population so far are described in (Table 2).

**Table 2:** Most frequently identified pathogenic mutations in the *BRCA* genes in Brazilian HBOC studies

Reference	T.2018	S.2014	F.2016	F.2014	E.2016	E.2011	L.2004	B.2017	B.2017	B.2020	M.2016	D.2005	C.2018	G.2007	C.2013	E.2009	C.2020	A.2017	M.2012	F.2018	G.2022	S.2021	M.2022	M. 2023	N.2021	E.2018	Total
Sample (n)	157	120	349	106	145	137	47	94	131	105	100	31	44	402	54	612	95	418	168	1380	1663	224	257	1267	49	79	
*BRCA1*																											
c.5266dupC	4	3	18			7	4	1	1	1	3	1	2	5	2	4	11	9			28	3		20		1	128
c.3331_3334delCAAG			8	4							3		1		1	1	1	1			13			14		1	49
c.211A > G				5					1	1											3		1	6		1	18
c.1687C > T			2							1	1							2			5	2	1	3			17
*BRCA2*																											
c.156_157insAlu			1		3												1	1	3	9	5	1	2	1			27
c.2808_2811delACAA		1	3												1		1	3			5		1	8		2	25
c.6405_6409delCTTAA			2							1				2				1			5	1		6			18
c.2 T > G			2										2					1			3		2	1	1		12

## Discussion

Brazil is one of the most populous countries in the world, and it has a great ethnic diversity that varies from state to state due to the colonization process that took place in the years 1500. According to the Brazilian Institute of Geography and Statistics (IBGE), in 2022 the Brazilian population reached 203 million inhabitants, making it the seventh most inhabited country in the world. Among the regions, the Southeast is the most populous, with about 85 million people. The Northeast has 54 million inhabitants and the South is home to approximately 30 million people. The North is the largest Brazilian region in terms of territorial extension (45% of the national territory), however, with about 17 million inhabitants. The Midwest region has a little more than 16 million inhabitants, making it the least populated region in the country. Regarding livelihood distribution, the Southeast and South regions have approximately twice the per capita household income of the North and Northeast regions [46, 47]; the latter also has fewer centers for molecular diagnosis and care for cancer patients [48].

The North region of Brazil appeared in only two study in this review, and the above data may partly explain why the vast majority of studies found were from the Southeast and South regions. Although we did not find other studies with populations from the North region, a previous study described reports of *BRCA1* and *BRCA2* tests from several Brazilian states, and 20 reports from the North region were described. The authors compiled the testing reports of probands with pathogenic/likely pathogenic variants referred to public and private health care centers distributed across 11 Brazilian States. They found two variants unique to this region [9]. This study was not targeted at the identification stage with the descriptors used and it was not clear whether part of the data presented had already been reported by previous primary studies. For this reason, it was not included in this review. Another study carried out in the north of the country found that three variants of the *BRCA2* gene, commonly linked to hereditary breast cancer, had a significantly higher allele frequency in Amazonian compared to other ethnic groups analyzed (Africans, Americans, Europeans and Asians) in control samples [49].

The first Brazilian study to include molecular evaluation of HBOC-related genes was published in 2004 by Lourenço et al. [34] and was based on screening only the *BRCA1* gene, followed by Dufloth in 2005 [26], who sequenced some exons of the *BRCA1* and *BRCA2* genes. The following studies, in subsequent years, also screened for point mutations or sequencing of some exons in *BRCA* genes [33, 35, 40, 43]. Only in 2013 the complete sequencing of the *BRCA* genes was conducted in Brazil by Carraro et al. [27], and other genes began to be explored in a punctual way [13, 18, 21]. From 2014 onwards, more works were published each year, and other genes related to HBOC were analysed more frequently, mainly *TP53* and *CHEK2*. Until 2014, Sanger sequencing was unanimous, although other screening techniques were also used in conjunction. The NGS platform was launched in 2005, and although the spectrum of articles found in this review ranges from 2004 to 2023, the first articles to use NGS for HBOC studies were published in 2016 [15, 19, 25]; however, none of them screened for other susceptibility genes. The first to study a panel including other genes were Timoteo et al. in 2018 [12], with 11 genes related to hereditary breast cancer, and the only one to carry out a multigene analysis in the Northeast region. Overall, most articles from 2020 to 2023 performed multigene screening in HBOC patients from Brazilian population. Only a single study used a more advanced technique, whole exome sequencing, to identify new genes related to breast and/or ovarian cancer [22].

There was high variability in the frequency of the mutations found among the 35 articles analyzed. The detection percentage of pathogenic mutations varied from 0.0% to 29.5% among the studies. These major differences may be due to the inclusion criteria used in each study, the genes evaluated, the wide variation in the techniques used and the prevalence of *BRCA* mutations among different ethnicities. Individuals who meet multiple criteria are more likely to carry a pathogenic variant in *BRCA* genes [14]. Works that performed the complete sequencing of *BRCA* genes found higher rates of mutations in the population [7, 18, 31], contrasting with those tracking for point mutations as expected [16, 30, 42]. Only one study performed whole-exome sequencing [22], and mutations were found in genes not reported in any other study.

The pathogenic mutation frequency was higher in the *BRCA1* than in the *BRCA2* gene in most of the studies that performed complete *BRCA* sequencing. In these works, the frequency of mutations found in both *BRCA* genes ranged from 0.5% to 17%. Only two studies, in Minas Gerais and São Paulo states reported a higher frequency of *BRCA2* mutations compared to *BRCA1* [29, 31]. Of the 35 studies analysed, 17 studies found pathogenic mutations in other genes, with a total of 28 mutated genes: *ATM*—*ATR*—*BARD1*—*BIRP1*—*BLM*—*CDH1*—*CHEK2*—*CTNNA1*—*CTNNA2—DROSHA*—*FANCC*—*HERPUD1*—*LCP1*—*MLH1*—*MRE11A*—*MSH2*—*MSH6*—*MUTYH*—*NBN PALB2*—*PLK2*—*PTCH1*—*PMS2*—*RAD51C*—*RAD51D*—*RECQL4*—*RIPK1* and *TP53*.

In this review, 23 studies described variants of unknown significance (VUS) in their results. These variants create an obstacle to patient diagnosis, since they raise doubts about their real role in the pathology of breast cancer. Different criteria have been adopted to indicate the pathogenicity of VUS as bioinformatics, frequency in a control population and functional studies. However, classification is not always possible. Although 23 articles reported the presence of VUS, we believe that others also found it but did not report it, mainly the works that used the NGS methodology. Therefore, a more in-depth study within the database of each work is necessary to expand our knowledge of VUS in Brazil.

After analyzing the database with pathogenic mutations most frequently identified in *BRCA* genes and excluding studies with overlapping samples, we can verify that the c.5266dupC mutation in *BRCA1* is the most commonly found in Brazil in patients with breast and/or ovarian cancer. The c.5266dupC mutation was first described as a founder effect in the Ashkenazi Jewish population [50, 51]. This mutation was found in 20 of 24 studies where it was investigated, and the highest frequencies found in Brazilian studies were 11.6% (11/95) followed by 8.5% (4/47) and 5.1% (7/137), all from the Southeast and South regions [7, 35, 41]. Although the mutation *BRCA1* c.5266dupC has been reported in different regions of Brazil, it is more frequently found in populations with Central and Eastern Europe ancestors [49]. Considering all studies that track this mutation in the *BRCA1* gene, we found a frequency of 2% (120/6008) for this mutation in Brazilian patients (Additional file 1). The results reported here are in accordance with Palmero et al. [9], where the c.5266dupC mutation was found as the most frequent mutation, representing to 20.2% of all variants found in the *BRCA1* gene in Brazil. In this review, this mutation corresponded to 26.8% of all pathogenic mutations found in the *BRCA1* gene.

The c.3331_3334delCAAG mutation was the second most frequent mutation found in the *BRCA1* gene, with a total of 49 records distributed among twelve studies. Represented in different populations, it was first described in Canadian families [52]. To know the origin of this mutation, a study conducted in 2020 by Tuazon and collaborators [53] performed haplotype analysis with populations from Colombia, Spain, Portugal, Chile, Africa and Brazil and suggested that this mutation had a single origin in the Iberian Peninsula and was introduced in Colombia and South America at the time of Spanish colonization. This mutation was responsible for 10.2% of *BRCA1* mutations found in Brazil in this review.

The c.211A > G mutation was the third most frequent in the *BRCA1* gene been responsible for 3.8% of mutations in this gene. It is a mutation of Spanish origin and consists of a Galician founder mutation [54]. The c.1687C > T mutation was found in eight different studies with samples from all regions of Brazil, being the fourth most reported variant in HBOC patients in the Brazilian population. It was identified with an allele frequency of 0.000006576 in the general population and 0.00001471 in the European (non-Finnish) population, by the Genome Aggregation Database (gnD: https://gnomad.broadinstitute.org/).

The most frequent mutation found in the *BRCA2* gene was c.156_157insAlu, although it was tracked by few studies. This mutation was classified as a founder due to the high frequency described in the Portuguese population [55]. The high occurrence of this mutation in Brazil possibly involves the arrival of Portuguese immigrants to Brazilian lands during centuries of colonization. The frequency of this mutation in HBOC patients from Brazilian studies varied from 0.007 to 2.06%. As many studies did not track this mutation, which requires a specific PCR reaction, and we have no information as to whether the studies that used MLPA included probes for this region, it was not possible to check their global frequency in HBOC patients from Brazil. This mutation was responsible for 9.6% of all pathogenic mutations reported in the *BRCA2* gene, and we strongly suggest that it be screened in Brazilian patients. The c.2808_2811delACAA mutation in the *BRCA2* gene was observed in nine Brazilian studies. It was the second most recurrent mutation in the *BRCA2* gene reported in 25 patients from different regions of the country. This mutation was described in seven different countries in Western Europe and North America, and it was considered a Norwegian founder mutation [56, 57].

The c.6405_6409delCTTAA mutation was the third most frequent in the *BRCA2* gene, reported in seven Brazilian studies. It was first described as 6630del5 in a study that tracked 25 families with breast and/or ovarian cancer cases in the UK and Ireland [58]. This mutation demonstrates an allele frequency in the global population of 0.000004156 in the gnomAD database.

The c.2 T > G mutation was the fourth most frequent mutation found in the *BRCA2* gene, with a total of twelve records in this review. It has been reported in Portuguese families [59] and published in population databases with a total allele frequency of 0.000007957 and 0.00005784 in a mixed Latin American population by gnomAD.

The most frequently mutated gene after *BRCA1/2* was *TP53*. The variant c.1010G > A (p.R337H) is a Brazilian founder mutation, identified in 0.3% of the general population in South Brazil [60]. Many other studies about this mutation have been conducted in the Brazilian population and were not included in this review because of the descriptors used here. This mutation is associated with a variety of cancers, most notably those of the Li-Fraumeni syndrome spectrum. However, several studies have associated the presence of this mutation with breast cancer and the HBOC syndrome [21, 61]. In a previous study with cancer patients and HBOC/HBCC patients from the South region, the mutation c.1010G > A was found with a frequency of 8.6% and 3.4%, respectively [62]. In this review, the frequency of this mutation ranged from 0.8% to 7.1% among HBOC patients. The highest frequency was identified in the study by Cury et al. [21] (2/28) and the lowest in Gomes et al. [36] (1/126). Of 16 studies that tracked the *TP53* gene, 13 found pathogenic mutations, and 11 of them found the c.1010G > A variant. Considering all studies that screened for this specific mutation, the frequency of c.1010G > A in patients who met clinical criteria for HBOC from Brazil was estimated in 1.83% (61/3336) (Additional file 1). This lower frequency, compared to that found by Giacomazzi et al. [62] in HBOC patients, could reflect the greater variability of populations studied in this review, as here, in addition to the South region, many other studies from other regions of Brazil were also included.

Although a greater number of studies in this review have focused their attention on the *CHEK2* and *ATM* gene, the second most mutated non-*BRCA* gene, curiously, was *MUTYH*. Ten different pathogenic variants were found in the *MUTYH* gene with a total of 34 mutated patients (Additional file 1), distributed in three studies [24, 38, 44]. In highlight, the c.1187G > A variant was reported in fifteen patients with HBOC in Brazil, including 13 patients from the cohort of Guindalini et al. [44], been this variant responsible for a significant proportion of pathogenic mutations in the *MUTYH* gene.

The third gene with the highest number of pathogenic variants reported in Brazil was *ATM*. Twenty-three pathogenic variants were reported in 32 HBOC patients (Additional file 1). Among nine studies using NGS technology, six found pathogenic mutations in the *ATM* gene, distributed in different regions of the country [12, 23, 36–38, 44]. Next comes the *PALB2* gene with 14 patients identified as carriers of the pathogenic mutation (Additional file 1).

Both the *ATM*, *PALB2* and *CHEK2* genes are breast cancer susceptibility genes of moderate penetrance. The *CHEK2* gene was tracked by NGS panel studies, and the most reported 1100delC mutation was also tracked punctually by five different studies [63]. However, of the 14 studies that included the *CHEK2* gene for screening in this review, only three were identified the c.1100delC variant [16, 18, 44]. This mutation has been described in less heterogeneous populations, as in studies conducted in the Netherlands that described the increased risk of breast cancer observed in patients with *CHEK2* 1100delC and another study that reported 4% of their tested patients carrying this pathogenic variant [64, 65]. Studies show that carriers with a family history of breast cancer are at 2–3 times greater risk when breast cancer is associated with the *CHEK2* c.1100delC mutation [66]. In Brazil, studies tracking mutations in moderate- or low-penetrance genes for breast cancer are still rare, but this number should increase in the next year with the advent of NGS technology.

## Limitations

Data analysis was challenging, and the review had some limitations, one of which was the scarcity of data from two regions of Brazil (the Midwest and the North). Although these are the least populous regions, their inclusion would give an interesting global view of the mutation profile. It was not possible to obtain information about the place of birth of the patients included in the studies. Therefore, data on geographic location should be interpreted with caution. In addition, some studies may not have been very representative of the region studied due to the sample size or study objective. Some works focused on tracking specific mutations or specific regions of the selected gene. The works are heterogeneous in their aim, which leads to an asymmetrical presentation of the results, leaving out some important data, such as VUS. The classification and final interpretation of the detected variants is also a limitation of this study, considering that many variants of uncertain meaning described mainly in older studies may currently lead to another classification based on the recommendations of the American College of Genetics and Medical Genomics (ACMG). It is important to highlight that this review did not aim to reclassify the variants in each study.

## Conclusion

The identification of germline mutations is a crucial factor in the continuation and updating of clinical management protocols. Despite significant molecular heterogeneity among mutations in HBOC patients from Brazil, three mutations deserve to be highlighted: c.5266dupC, c.156_157insAlu and c.1010G > A in the *BRCA1*, *BRCA2* and *TP53* genes, respectively. With more than 200 records, these mutations play a key role in the pathology of breast cancer in Brazil. In addition, it was seen that *MUTYH*, *ATM*, *PALB2* and *CHEK2* genes also contributed significantly to the increased risk in HBOC patients. However, the accumulated knowledge about mutation profiles in HBOC susceptibility genes do not cover all Brazilian subpopulations, especially those from the Amazon in the north of the country. The multigene panels contributed to a greater identification of pathogenic mutations in non-*BRCA1/2* genes, but the number of studies is not yet sufficient to show the full spectrum of mutations in the country.

### Supplementary Information

**Additional file 1: **Shows all pathogenic mutations identified in the reviewed articles and the estimated frequencies.

## Data Availability

All data from this research are already available in the suplemmentary material.

## Abbreviations

ACMG: American College of Genetics and Medical Genomics
AS-PCR: Allele-specific PCR
BC: Breast cancer
DGGE: Denaturing gradient gel electrophoresis
DHPLC: Denaturing high performance liquid chromatography
gnomAD: Genome Aggregation Database
HBCC: Breast and colorectal cancer syndrome
HBOC: Hereditary breast and ovarian cancer syndrome
HRM: High resolution melting
IBGE: Brazilian Institute of Geography and Statistics
LFL: Li-Fraumeni Syndrome
MLPA: Multiplex ligation-dependent probe amplification
NGS: Next-generation sequencing
PPT: Protein Truncation Test
qPCR: Real-time PCR
RFLP: Restriction fragment length polymorphism
SSCP: Single-Stranded conformation polymorphism
VUS: Variants of unknown significance
WES: Whole exome sequencing
